# Treatment of hepatic encephalopathy by on-line hemodiafiltration: a case series study

**DOI:** 10.1186/1471-227X-10-10

**Published:** 2010-05-21

**Authors:** Shinju Arata, Katsuaki Tanaka, Kazuhisa Takayama, Yoshihiro Moriwaki, Noriyuki Suzuki, Mitsugi Sugiyama, Kazuo Aoyagi

**Affiliations:** 1 Critical Care and Emergency Center and Gastroenterological Center, Yokohama City University Medical Center, 4-56 Urafune-cho, Minami-ku, Yokohama, Japan

## Abstract

**Background:**

It is thought that a good survival rate of patients with acute liver failure can be achieved by establishing an artificial liver support system that reliably compensates liver function until the liver regenerates or a patient undergoes transplantation. We introduced a new artificial liver support system, on-line hemodiafiltration, in patients with acute liver failure.

**Methods:**

This case series study was conducted from May 2001 to October 2008 at the medical intensive care unit of a tertiary care academic medical center. Seventeen consecutive patients who admitted to our hospital presenting with acute liver failure were treated with artificial liver support including daily on-line hemodiafiltration and plasma exchange.

**Results:**

After 4.9 ± 0.7 (mean ± SD) on-line hemodiafiltration sessions, 16 of 17 (94.1%) patients completely recovered from hepatic encephalopathy and maintained consciousness for 16.4 ± 3.4 (7-55) days until discontinuation of artificial liver support (a total of 14.4 ± 2.6 [6-47] on-line hemodiafiltration sessions). Significant correlation was observed between the degree of encephalopathy and number of sessions of on-line HDF required for recovery of consciousness. Of the 16 patients who recovered consciousness, 7 fully recovered and returned to society with no cognitive sequelae, 3 died of complications of acute liver failure except brain edema, and the remaining 6 were candidates for liver transplantation; 2 of them received living-related liver transplantation but 4 died without transplantation after discontinuation of therapy.

**Conclusions:**

On-line hemodiafiltration was effective in patients with acute liver failure, and consciousness was maintained for the duration of artificial liver support, even in those in whom it was considered that hepatic function was completely abolished.

## Background

Acute liver failure is usually fatal, and in the US, the mortality rate remains as high as 30% in adult patients [1]. Although liver transplantation for acute liver failure has increased the survival rate markedly, the limited supply of suitable livers implies that transplantation is not available to all patients; 22.7% of patients who were listed for transplantation die awaiting transplantation 3 days after being listed (range 1-6 days). On the other hand, 45% of patients with acute liver failure survive with medical therapy alone without liver transplantation [1]. It is thought that a good survival rate can be achieved by establishing an artificial liver support (ALS) system that reliably compensates liver function until the liver regenerates or a patient undergoes transplantation. Although various attempts have been made to develop an ALS system, there is no consensus about the best method [2,3].

A combination of hemodiafiltration (HDF) and plasma exchange has been frequently used in patients with acute liver failure in Japan, and this method results in improvement of consciousness in 90% or more of cases [4]. However, HDF is complex and is not available in general facilities. Moreover, there are economical problems, the practical therapy is often shortened or limited in efficiency because of the cost. Infusion-free HDF "on-line HDF," was developed with the purpose of solving these problems [5]. On-line HDF markedly reduces the cost of blood purification therapy and simplifies the setup of the dialysis monitor. A number of clinical benefits of this method in the treatment for chronic renal failure were reported, especially the superior efficiency removing middle molecules [6-12].

We introduced a new ALS system, on-line HDF, in patients with acute liver failure. Here, we report our experience in a substantial number of patients with acute liver failure treated with this ALS system.

## Methods

### Patients

	Between May 2001 and October 2008, 25 patients with acute liver failure were treated at our hospital. Acute liver failure was diagnosed by the presence of coagulopathy (prothrombin time [PT] international normalized ratio [INR] ≥1.5), any degree of hepatic encephalopathy, and length of illness ≤24 weeks [1]. Acute liver failure was also confirmed by the medical history, clinical findings, biochemical test, viral serologies, and imaging methods. The exclusion criteria for entry into the study were: 1) clinical evidence of severe cerebral edema or cerebral herniation at the time of admission, 2) no consent to on-line HDF by patient or relatives, and 3) obvious improvement of condition at the time of hospitalization. Eight patients with acute liver failure admitted during the study period were excluded from the analysis on the basis of these exclusion criteria. Three patients presented deep coma, and severe cerebral edema at the time of admission. All these 3 patients also presented multiple organ failure, and died 1, 2, and 4 days after admission, respectively. In a patient who presented hypovolemic shock due to dehydration, we could not obtain the consent because he did not have relatives, and standard medical therapy improved his consciousness promptly. In the remaining 4 patients who presented acute liver failure due to congestion, the treatment for congestive heart failure improved their condition with no need of ALS. Ultimately 17 patients were included in this study. The characteristics of the participating patients (baseline clinical and laboratory data) are summarized in Table 1. There were 11 men, aged 26-72 years (49.3 ± 4.3 years), and 6 women, aged 21-52 years (40.7 ± 4.5 years). The etiology of acute liver failure was hepatitis B virus infection in 10 patients, non-A~G hepatitis virus infection in 2 (indeterminate), alcoholic suspected with the medical history in 2, congestive liver in 1, infiltration of leukemia cells in 1, and acetaminophen overdose in 1. In eight patients of 10 patients who suffered from hepatitis B virus infection, the hepatitis B surface antigen and an IgM antibody to the hepatitis B core antigen were positive (acute infection). In the remaining 2 patients, the medical history that they had been healthy carriers of hepatitis B virus was proven by their medical records, and viral serologies on admission revealed acute exacerbation of hepatitis B infection. Acute liver failure developed in a patient of these 2 patients after the interruption of administration of steroids for multiple myeloma. The average time from the onset of the disease until admission to the hospital was 10.4 ± 3.3 days with a range of 3-60 days. Eleven of the 17 patients had encephalopathy on admission, and the remaining 6 had encephalopathy 2.7 ± 0.9 days after admission with a range of 1-6 days.

**Table 1:** Characteristics of participating patients who underwent artificial liver support with on-line hemodiafiltration.

Case	Gender	Age	Etiology	Onset-admission(days)	Onset-encephalopathy (days)	Highest AST (IU/L)	Laboratory findings at the start of on-line HDF			
							
							AST (IU/L)	TBil (mg/dL)	PT (INR)	ammnoia (μg/dL)
1	F	41	HBV acute infection	6	6	9280	1852	7.8	4.22	85
2	M	54	HBV carrier	17	19	6200	4470	5.6	4.52	116
3	M	53	HBV acute infection	4	5	6430	29	23.3	4.02	102
4	M	72	Congestive liver	5	5	11800	94	20.9	1.5	41
5	M	56	Leukemia	14	14	6584	6584	10	1.84	79
6	M	26	HBV acute infection	10	10	275	275	9	3.26	318
7	F	21	Indeterminate	3	3	25400	3295	7.4	3.45	107
8	M	48	HBV acute infection	6	7	11000	1690	4.7	2.79	157
9	F	36	Acetaminophen	4	4	5450	5450	4.6	12<	306
10	M	68	HBV acute infection	6	6	1259	187	6.6	2.06	294
11	M	43	Alcohol	3	9	20548	10570	4.5	2.15	253
12	M	40	HBV acute infection	5	6	8056	1023	11.5	2.44	223
13	M	53	HBV acute infection	7	6	8790	2765	9.3	2.65	246
14	M	29	HBV acute infection	5	5	902	678	16.1	12<	223
15	F	48	Indeterminate	60	65	227	88	6.4	2.95	155
16	F	46	Alcohol	9	8	84	81	15.8	1.6	302
17	F	52	HBV carrier	13	13	126	126	19.6	2.59	131

HBV, hepatitis B virus; HDF, hemodiafiltration; AST, asparatate aminotransferase; TBil, total bilirubin; PT, prothrombin time; Highest AST, Highest values that could be recorded during illness.

These patients were initially treated with intensive medical therapy, including immunosuppressive therapy, antiviral therapy (lamivudine or entecavir for hepatitis B virus infection), and the new ALS system comprising on-line HDF and plasma exchange. At the time of the diagnosis of acute liver failure was made, the need for liver transplantation was explained to the patient's relatives. Hepatic encephalopathy was assessed using the West Haven criteria of altered mental state [13] and the Glasgow Coma Scale in accordance with the recommendation of a working party on studies in hepatic encephalopathy [14]. Two investigators, who had extensive clinical experience in hepatic encephalopathy, confirmed all cases of encephalopathy by reviewing the clinical course of the patient during hospitalization. All patients underwent computerized tomography (CT) examination to determine liver volume on admission and at least once a week thereafter.

The study was performed according to the guidelines of the Declaration of Helsinki and the study protocol was approved by the ethics committee at our institution. Written informed consent was obtained from each patient or their relative if the patient was unable to give consent.

### Artificial liver support

#### On-line HDF

**Figure 1:**
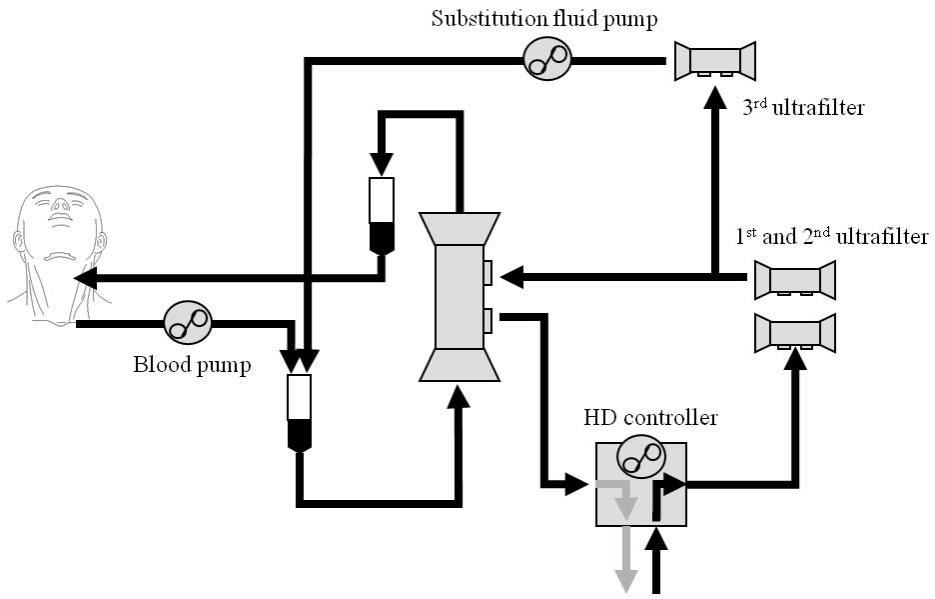
**The circuit of on-line hemodiafiltration with pre dilution**. Sterile substitution fluid produced on-line from the dialysate through three ultrafilters. Substitution fluid was infused pre-filter with a substitution fluid pump. HD, hemodialysis.

Blood access was established with a double-lumen catheter (Vas-Cath^®^, Niagara^®^; Bard, Salt Lake City, UT, USA) inserted into a central vein with an internal jugular vein approach. On-line HDF was performed as previously described [15,16]. In brief, in the on-line solution preparation system, substitution fluid was prepared continuously by ultrafiltration of dialysate, enabling its use as substitution fluid. In our on-line system, two ultrafilters (EF-01, FLX-18GW, polyester-polymer alloy [PEPA] membranes; Nikkiso, Tokyo, Japan) were used for cold sterilization of the dialysate (AK-Solita^® ^FL; Ajinomoto Pharma, Tokyo, Japan) in hemodialysis (HD), and one ultrafilter (EF-01, FLX-18GW, PEPA; Nikkiso) was added when using substitution fluid (Figure 1). Sterile substitution fluid produced on-line from the dialysate was infused pre-filter with a substitution fluid pump and tubing set (PRS-12, NV-A300PA; Nikkiso) (pre-dilution). HDF was performed using filters containing 1.5 m^2 ^of polysulfone membranes (APS-15E; Asahi Kasei Medical Co., Tokyo, Japan). Pore size was 85 Å. A HD control device and tubing set (model DBG-02, NV-Y888PC; Nikkiso) were used. An AK-Solita FL was set to prepare 700 mL/min of dialysate. Substitution fluid flow rates ranged from 300 to 350 mL/min, so that actual dialysate flow rates during HDF ranged from 350 to 400 mL/min. Blood flow rates ranged from 300 to 350 mL/min. At the start, the duration of on-line HDF was set so that the amount of hemocatharsis (blood flow rate × time) was three times the estimated body fluid volume (actual body weight ×0.6). If the patient recovered from encephalopathy and disorientation disappeared, the duration of on-line HDF was reduced to two-thirds of the initial duration, and if consciousness was maintained under this condition, on-line HDF was given once per 2 days.

#### Plasma exchange

In general, plasma exchange was performed using 40 packs of fresh frozen plasma per treatment. If PT-INR on the day after previous plasma exchange was higher than 2.0, plasma exchange was performed with the same setting. If PT-INR was less than 2.0, infusion of 8 packs of fresh frozen plasma was performed in place of plasma exchange. During infusion of fresh frozen plasma, we reduced the quantity of the other infusion solution to avoid fluid overloading.

### Statistical Analysis

Data are expressed as mean ± SD. We performed simple linear regression analysis to determine whether the degree of encephalopathy (stage of hepatic encephalopathy and Glasgow Coma Scale), patient's age, asparatate aminotransferase, total bilirubin, PT, and ammonia at the start of on-line HDF was associated with the number of sessions of on-line HDF from the start of the treatment to recovery of consciousness. We used student's *t*-test to compare between continuous data of patients who survived hepatic failure without transplantation and those of patients who died of hepatic failure. All P values were two-sided, and values less than 0.05 were considered statistically significant.

## Results

Tolerance to on-line HDF was excellent, pyrogenic reactions, bleeding complications, progress of coagulopathy that the treatment related to were not observed. The consciousness level, summary of ALS, and prognosis of participating patients who underwent ALS with on-line HDF are summarized in Table 2. At the start of the study, 2 patients had stage 2 encephalopathy, 7 had stage 3, and 8 had stage 4. During ALS with on-line HDF, 1 patient (Case 6) withdrew from the further investigation. He suffered from brain herniation with rapid progression of cerebral edema on the first day of admission and fell into deep coma. A flat wave was confirmed by electroencephalography performed on the second day. The prolongation of the treatment was thought to be not worthwhile. ALS was discontinued after we obtained his family's consent. He died on the 5th day of hospitalization. However, all the remaining 16 patients recovered consciousness after ALS with on-line HDF, and the average number of sessions of on-line HDF from the start of the treatment to recovery of the patient's consciousness was 4.9 ± 0.7 with a range of 1-10. The average total number of sessions of on-line HDF was 14.4 ± 2.6 with a range of 6-47 over a period of 7-55 days (mean 16.4 ± 3.4 days). During ALS with on-line HDF, plasma exchange was performed in 11 patients with a range of 2-17 sessions (mean 7.2 ± 1.3 sessions).

**Table 2:** The consciousness level, summary of ALS, and outcome of participating patients who underwent artificial liver support with on-line hemodiafiltration.

Case	The degree of HE at the start of on-line HDF		On-line HDF to recovery of consciousness (sessions)	Total on-line HDF (sessions)	Plasma exchange (sessions)	Duration of ALS (days)	Outcome
						
	GCS	Stage*					
1	8	4	5	12	7	13	Spontaneous survival
2	13	3	2	11	9	13	Death (no donor to transplant)
3	13	2	1	15	10	15	Death (no donor to transplant)
4	9	4	7	7	0	7	Death due to heart failure
5	11	3	4	15	0	15	Death due to sepsis
6	4	4	-	5	4	5	Death due to cerebral herniation
7	12	4	5	8	2	10	Spontaneous survival
8	13	3	8	47	5	55	Spontaneous survival
9	10	4	7	21	8	24	Death from respiratory failure
10	6	4	7	14	9	14	Death (no donor to transplant)
11	14	2	1	9	2	9	Spontaneous survival
12	13	3	1	13	2	13	Spontaneous survival
13	13	3	5	7	0	7	Spontaneous survival
14	6	4	10	27	17	42	Death (no donor to transplant)
15	12	3	5	7	0	7	Transplantation
16	4	4	5	6	0	7	Spontaneous survival
17	8	3	6	11	8	12	Transplantation

HE, hepatic encephalopathy; HDF, hemodiafiltration; GCS, Glasgow Coma Scale; ALS, artificial liver support. *Hepatic encephalopathy staging by the West Haven criteria of altered mental state [13].

Of the 16 patients who recovered consciousness, 7 recovered without transplantation (spontaneous survival) and 3 died of congestive heart failure, sepsis, or respiratory failure. Two underwent living-related liver transplantation. The remaining 4 patients were candidates for liver transplantation, but these 4 patients died without transplantation because of the lack of a living donor candidate. In patients who were candidates for transplantation but died without it, on-line HDF was performed in 11-27 sessions (mean, 16.8 ± 3.5 sessions) and plasma exchange was performed in all patients, with 9-17 sessions (mean 11.3 ± 1.9 sessions) over a period of 13-42 days (mean 21.0 ± 7.0 days). During ALS with on-line HDF, these patients showed clear consciousness; however, they died of severe hepatic failure 2-4 days after the termination of intensive medical care. Final liver volumes, estimated by CT or proven by autopsy, ranged from 332 to 467 mL (mean 375.0 ± 31.5 mL). Autopsy specimens from 1 patient revealed no sign of regeneration of the liver pathologically.

**Figure 2:**
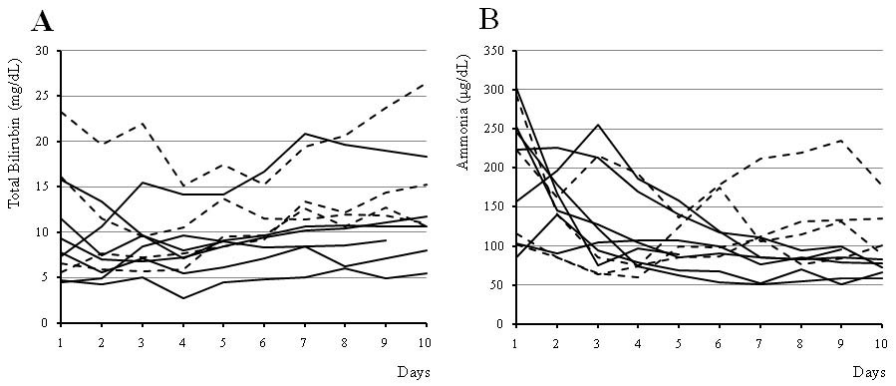
**The changes of the serum bilirubin and ammonia levels during first ten days after the start of artificial liver support**. The solid line and dashed line shows the values of the patients who survived hepatic failure without transplantation (Case 1, 7, 8, 11-13, 16) and the patients who died of liver failure (Case 2, 3, 10, 14), respectively. The serum bilirubin levels increased gradually in almost patients of both groups (A). The serum ammonia levels of the patients who survived hepatic failure without transplantation decreased to less than 100 μg/dL on the 7th day after the start of the treatment with a constant tendency (B).

Figure 2 shows the changes of serum bilirubin and ammonia levels during first ten days after the start of ALS dividing it into two groups of the 7 patients with spontaneous survival and the 4 patients who died of liver failure. The serum bilirubin levels increased gradually in both groups, whereas the serum ammonia levels of the patients with spontaneous survival decreased to less than 100 μg/dL on the 7th day after the start of the treatment with a constant tendency.

**Figure 3:**
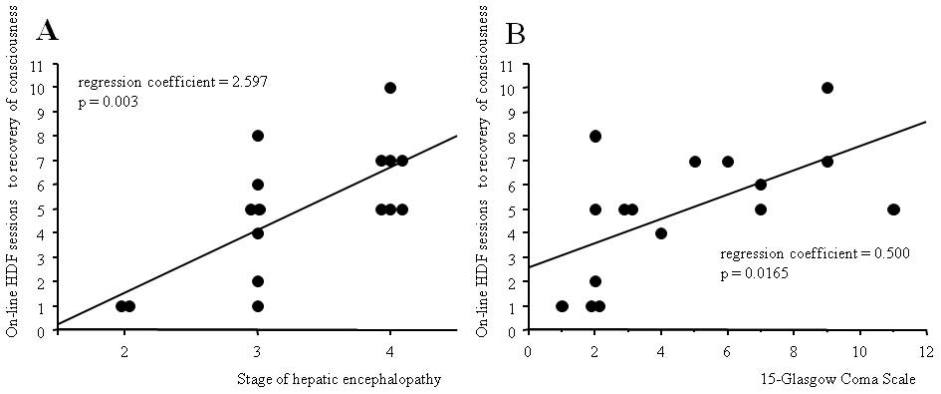
**Correlation between the degree of encephalopathy and the number of sessions of on-line hemodiafiltration to recovery of consciousness**. Significant correlation was observed between (A) stage of hepatic encephalopathy [13] and (B) Glasgow Coma Scale at the start of on-line hemodiafiltration and the number of sessions of on-line hemodiafiltration from the start of the treatment to recovery of consciousness. HDF, hemodiafiltration.

Significant correlation was observed between the degree of encephalopathy (stage of hepatic encephalopathy and Glasgow Coma Scale) at the start of on-line HDF and the number of sessions of on-line HDF from the start of the treatment to recovery of consciousness (Figure 3). However, no significant correlation was observed between the number of sessions of on-line HDF from the start of the treatment to recovery of consciousness and the following parameters: patient's age, asparatate aminotransferase, total bilirubin, PT, and ammonia at the start of on-line HDF. There were also no significant differences between 7 patients who survived hepatic failure without transplantation and 4 patients who died of hepatic failure with respect to the average time from disease onset to hospital admission, and the number of sessions of on-line HDF from the start of the treatment to recovery of consciousness.

### Case presentation

#### Case 14

**Figure 4:**
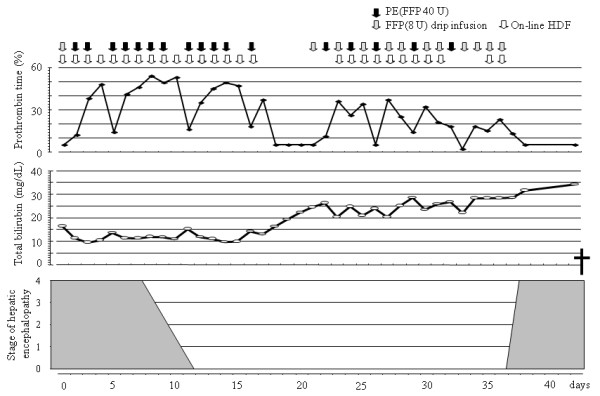
**Clinical course after the start of artificial liver support with on-line hemodiafiltration**. After 10 on-line hemodiafiltration sessions, a 29-year-old man with acute hepatitis B virus infection (Case 14) experienced complete and rapid resolution of hepatic encephalopathy. Artificial liver support with on-line hemodiafiltration and plasma exchange maintained lucid consciousness and minimal coagulation function for 25 days during an aggressive treatment period. Prothronbin time deteriorated without the plasma exchange rapidly and it could not be maintained by 8 units drip infusion of fresh frozen plasma. Computerized tomography examination on 16th day revealed further progression of liver atrophy, liver transplantation was therefore recommended but he could not receive liver transplantation because of the lack of a living donor candidate. Then, the treatment was reduced, and discontinued on the 36th day. He died on the 42nd day. PE, plasma exchange; FFP, fresh frozen plasma; HDF, hemodiafiltration.

**Figure 5:**
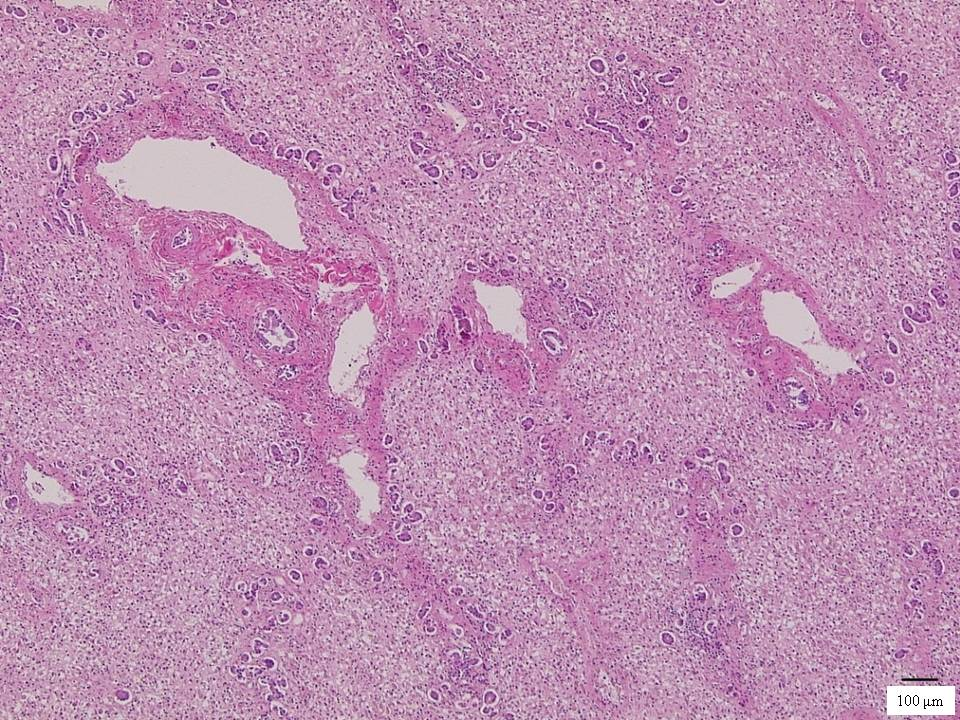
**Photomicrograph of histopathological specimen**. Histopathological specimen (hematoxylin and eosin staining) obtained from case 14 revealed absence of hepatocytes and destruction of normal structure and inflammatory cell infiltration. Portal venous areas are close each other and hyperplasia of the small bile ducts is seen.

Five weeks after completion of a tattoo procedure on his back, a 29-year-old Japanese man was transferred to our hospital for treatment of acute liver failure due to acute hepatitis B virus infection. At admission, his consciousness level represented stage 4 encephalopathy (Glasgow Coma Scale E1V1M4). Liver volume estimated by CT was 650 mL. Figure 4 depicts the clinical course after the start of on-line HDF. He became responsive to calling and completely recovered from encephalopathy after 10 daily sessions of on-line HDF. On the 13th hospital day, oral intake was started and his consciousness remained clear with the scheduled on-line HDF. CT examination revealed further progression of liver atrophy and liver transplantation was therefore recommended to the patient and his relatives; however, the relatives refused. He died on the 42nd hospital day from severe hepatic failure; however, his consciousness remained clear until discontinuation of ALS with on-line HDF. Autopsy 19 h after death revealed a liver weight of 332 g and the absence of viable hepatocytes (Figure 5).

#### Case 17

A 52-year-old Chinese woman had been diagnosed as a healthy carrier of Hepatitis B Virus at the time of previous orthopedic treatment. Her illness began with general fatigue and appetite loss on the day before hospital admission. Acute liver failure was diagnosed and she was started on steroid injections, entecavir, and fresh frozen plasma supplementation for 12 days, but disturbance of consciousness appeared and she was transferred to our hospital. At admission, her consciousness level represented stage 3 encephalopathy (Glasgow Coma Scale E2V2M4). CT examination revealed marked liver atrophy. Daily on-line HDF and PE were promptly started. She completely recovered from encephalopathy after six daily sessions of on-line HDF. Her consciousness remained clear with daily on-line HDF. On the 10th hospital day, CT revealed no sign of liver regeneration, and the estimated liver volume was 592 mL. Liver transplantation was performed and the weight of her extracted liver was 700 g.

## Discussion

We introduced ALS using on-line HDF with plasma exchange for patients with acute liver failure. In our experience, all patients, except one died of cerebral herniation with rapid progression of severe cerebral edema on the first day of admission, recovered consciousness after 4.9 ± 0.7 sessions of on-line HDF. Furthermore, consciousness remained clear after initial improvement by ALS with on-line HDF over a period of 16.4 ± 3.4 days until discontinuation of treatment.

Unusual accumulation of agents acting on the central nervous system requiring the liver to deal with toxic substances was the main mechanism of consciousness disorder caused by liver failure [17]. Ammonia, a key toxin in these substances [18], is related to brain edema and may lead to cerebral herniation, which is a major cause of death in patients with acute liver failure. An arterial blood ammonia concentration above 200 μg/dL conferred a high risk of cerebral herniation [19]. On the other hand, our experience is that serum ammonia concentration does not correlate with the degree of hepatic encephalopathy in patients with acute liver failure occasionally. It was shown that HD was insufficient for the treatment of hepatic encephalopathy [20], although it could remove ammonia, a small molecule [21-23]. At present, it is proposed as one opinion that the causal agents of hepatic encephalopathy are presumed to be middle molecules [24]. Blood purification therapy for patients with acute liver failure aims to remove ammonia, which can cause a critical situation with brain edema, and middle molecules have a high potential for central nervous system toxicity.

Splendiani et al. [20] reported improvement of consciousness in 37.5% of patients with acute liver failure who were treated with plasmapheresis. Therefore, plasma exchange alone is clearly insufficient for maintaining alert wakefulness in patients with severe hepatic encephalopathy. Improvement of consciousness in patients with hepatic encephalopathy was reported in 40% of those treated with HD and 78% of those receiving hemofiltration (HF) [20]. HD is effective in removing substances of small molecular weight but cannot provide efficient removal of substances of middle molecular weight. HF is effective in removing middle molecular weight substances but cannot remove small molecules effectively [25]. To compensate for these disadvantages, HDF is widely acknowledged today as a means of removing both small and middle molecular weight substances in renal replacement therapy [26-28].

In HDF, there are costs and storage problems because of the large amount of sterile substitution fluid required, which is usually supplied in ready-to-use bags. Furthermore, there is the need to connect multiple bags and tubing segments, the circuit is relatively complicated, and the risk of blood contamination may be high. For these reasons, HDF has not been applied routinely in the treatment of chronic renal failure, and is not commonly available in general facilities for the treatment of acute liver failure.

The new technique of on-line HDF is superior to conventional HDF and reduces the cost and simplifies the procedure [5]. On-line HDF can supply an unlimited amount of substitution fluid because the dialysate is prepared using an on-line system. Previous studies which described efficacy of hemodiabsorption or hemofiltration also reported some beneficial effects on hepatic encephalopachy, but failed to demonstrate sufficient improvement of hepatic encephalopathy [20,29,30]. Our ALS is much different from these studies at the point of amount of substitution fluid. Compared with HD, the only additional costs are those of the minute particle filter and the cost of controlling water quality. This system reduces the cost of substitution fluid and simplifies the setup of the dialysis monitor. Studies comparing it with high-flux HD reported its safety and superiority in the efficiency of removing middle molecules [6,7]. Furthermore, clinical findings of improved cardiovascular stability [8,9], reduced erythropoietin requirements [10,11], and improved immune response [12] with on-line HDF were reported. These were considered to be due to the removal of middle molecules with cardiodepressive effects, inhibitory effects on erythropoiesis, or depressive effects on the immune system.

The purification of the supplied water and the control of line cleaning make it possible to give the patient dialysate as a substitution fluid. The 1994 water quality standard in Japan entailed an obligation to use at least two ultrafilters after the dialysate adjustment device for cold sterilization. In studies of on-line HDF, the quality of the dialysate after the first ultrafilter met European Pharmacopoeia and US Pharmacopoeia standards for large-volume substitution fluid, i.e., no detectable bacteria and an endotoxin concentration below 0.25 IU/mL [31], and the microbiological quality of the dialysate after second ultrafilter was not different from that of autoclaved isotonic saline solution [32].

Infusion of hypertonic saline to maintain serum sodium levels of 145-155 mEq/L reduced the incidence and severity of intracranial hypertension in patients with grade 3 or 4 hepatic encephalopathy [33]. Like reducing ammonia, the correction of hyponatremia, a common electrolyte disorder in acute liver failure, is important for the avoidance of the early death due to brain herniation. Use of an on-line HDF system made it possible to vary the composition of dialysate individually by choosing different concentrates of electrolytes and machine settings. In this study we adjusted the sodium concentration of dialysate to 142-154 mEq/L to maintain the serum sodium level at 142 mEq/L or more. Only one of the study patients progressed to brain herniation during treatment.

**Figure 6:**
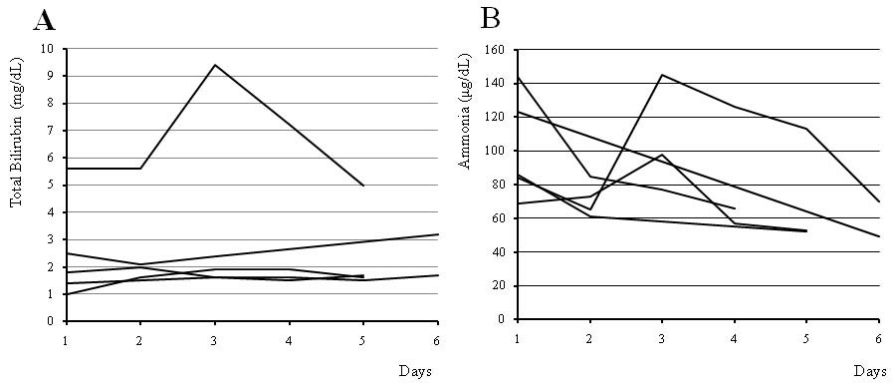
**The changes of the serum bilirubin and ammonia levels during first six days after the start of the standard medical therapy**. The solid line shows the changes of the serum bilirubin and ammonia levels in 5 patients who were excluded from the study because of improvement with no need of ALS. The serum bilirubin levels increased gradually in some patients (A). The serum ammonia levels decreased to less than 100 μg/dL on the 6th day after the start of the standard medical therapy (B).

Figure 6 shows the changes of the serum bilirubin and ammonia levels in 5 patients who were excluded from the study because of improvement with no need of ALS. The serum bilirubin levels increased in some patients even during the good clinical course, whereas the serum ammonia levels decreased rapidly. Figure 2 and 6 suggested that the change of serum ammonia levels may be a more useful predictor of survival compared with that of serum bilirubin levels.

In the present study, a significant correlation was observed between the degree of encephalopathy at the start of on-line HDF and the number of sessions of on-line HDF from the start of the treatment to recovery of consciousness. The degree of encephalopathy at the start of on-line HDF may predict the number of sessions of on-line HDF needed for recovery of consciousness. Patients with severe hepatic encephalopathy at the start of on-line HDF may need more than 10 sessions of on-line HDF to recover. On the other hand, if patients with low grade hepatic encephalopathy do not recover consciousness after five or more sessions of on-line HDF, brain CT should be performed to evaluate edema or hemorrhage.

The excellent clearance of various molecular substances with on-line HDF results in a number of clinical benefits in treatment for chronic renal failure [8-12] and is probably also of value in patients with acute hepatic failure. On the other hand, efficiency of clearance often conflicts with selectivity. In a small series of observations, we found that albumin was removed at the rate of 3.9-8.8 g per on-line HDF session, necessitating compensation for the loss of albumin with appropriate plasma exchange. Furthermore, on-line HDF may remove unknown factors that promote liver regeneration. It is still controversial whether ALS may retard the rate of regeneration [34]. An appropriate frequency of on-line HDF should be chosen for patients with acute liver failure.

In meta-analysis of artificial and bioartificial support system for the acute liver failure fails to reduce mortality, but it shows some improvement of hepatic encephalopathy in comparison with the standard medical therapy [35]. In more recent randomized controlled trials, Hassanein et al reported that 5 days treatment with extracorporeal albumin dialysis using molecular adsorbent recirculating system is effective in 62% of cirrhotic patients with severe hepatic encephalopathy [36]. This system thought to be one of hopeful methods. However, 40% of the patients who treated with the standard medical therapy alone also improved their hepatic encephalopathy by 2 grades from baseline, and 34% of the patients whose hepatic encephalopathy did not respond to the any treatment survived after 2 weeks. There is a possibility that their experience cannot be just applied to the patients with acute liver failure. Our study was not controlled study and study population was small. A larger and randomized controlled trial is needed to confirm that our experience can be generalized.

## Conclusions

ALS with on-line HDF was effective in patients with acute liver failure; in our experience of 16 patients, the patient's consciousness could be maintained as long as the duration of ALS even in conditions in which hepatic function was considered to be completely abolished. Although further investigation is necessary to clarify whether the new ALS system improves the rate of spontaneous survival, this system may provide sufficient time to prepare for transplantation in patients with acute liver failure.

## Competing interests

The authors declare that they have no competing interests.

## Authors' contributions

SA and KTan contributed to conception and design, carried out data acquisition, analysis and interpretation, and drafted the manuscript. KTak contributed to data analysis and interpretation, and drafted the manuscript. YM and NS participated in drafting the manuscript. MS revised the manuscript critically. KA contributed to conception and design, supported blood purification technically. All authors read and approved the final manuscript.

## Pre-publication history

The pre-publication history for this paper can be accessed here:

http://www.biomedcentral.com/1471-227X/10/10/prepub
